# Cameron Lesions With Severe Anemia: A Case Report

**DOI:** 10.7759/cureus.50678

**Published:** 2023-12-17

**Authors:** Mathangi Rajaram-Gilkes, Juan J Cardona, Aishwarya J Gilkes

**Affiliations:** 1 Department of Medical Education, Geisinger Commonwealth School of Medicine, Scranton, USA; 2 Department of Neurosurgery, Tulane University School of Medicine, New Orleans, USA; 3 Internal Medicine, Saint George's University School of Medicine, Saint George, GRD

**Keywords:** esophagogastroduodenoscopy, microcytic hypochromic anemia, cameron lesions, hiatus hernia, upper gastrointestinal bleeding

## Abstract

This case presentation involves an elderly patient presenting with signs of severe anemia. Investigations lead to the detection of Cameron lesions within a large paraesophageal hiatus hernia (HH). These lesions have been described in the literature as being an incidental finding within the herniated stomach during endoscopy in patients with large HH who presented with microcytic hypochromic anemia. Progressive information regarding the relationship of this occurrence in patients with this specific type of anemia associated with HH has heightened physician awareness to rule out these lesions as a primary cause of chronic bleeding. There has been sporadic publication in literature stating Cameron lesions to be an unusual cause of chronic blood loss resulting in microcytic hypochromic anemia. Perhaps due to the lack of adequate emphasis on this frequent finding in elderly with HH in literature, textbooks are yet to include this condition as a differential diagnosis as one of the causes of upper GI bleeding (UGIB). This case study makes us ponder if this etiology is not rare after all and emphasizes the importance of considering Cameron lesions to be one of the established causes of chronic blood loss of upper GI origin in elderly with a large HH. Screening methods such as chest X-rays (CXR) could be used for early detection of the condition, and an esophagogastroduodenoscopy (EGD) for confirmation before requiring additional invasive investigations.

## Introduction

In most textbooks, the description of the gastrointestinal tract begins with the oral cavity and extends to the description of the esophagus, stomach, and rest of the tubular system. Among the different clinical scenarios that can cause upper gastrointestinal bleeding (UGIB), textbooks usually investigate conditions from the lower esophageal regions to the proximal small intestine. The most common etiologies mentioned are esophageal varices, peptic ulcer disease, esophagitis, angioectasia, and vascular lesions [1]. Hiatus hernia (HH) is not commonly mentioned as a differential diagnosis to cause UGIB with a threatening massive reduction in hemoglobin. Cameron and Higgins described lesions within the mucosa of the stomach that had herniated within the chest cavity, especially in paraesophageal HH. They named these as "Cameron lesions" in 1986. They studied chest X-rays (CXR) of patients who exhibited a soft tissue shadow within the chest cavity, which was suggestive of HH and performed an endoscopy on them. They described these lesions to be linear erosions or ulcerations in the gastric mucosa, which were commonly located close to the diaphragm and were predominantly found in patients with large HH. A diameter of more than three centimeters of the hernia within the chest cavity is considered a large HH [1]. In 1976, a retrospective study on endoscopic findings in sixteen patients who had upper UGIB exhibited HH with gastric ulcers in the mucosa. Studies done prior to 1986 state that conservative management of such patients had led to complete healing, but no further descriptions have been mentioned in literature until Cameron and Higgins described them [2]. Since then, intermittent publications indicate the discovery of Cameron lesions during esophagogastroduodenoscopy (EGD), especially in patients with HH, who most frequently (50%) presented with anemia [1]. The following case presentation brings to light the importance of this occurrence, narrowing down the possibility of these lesions to be the etiology of microcytic hypochromic anemia.

## Case presentation

A 79-year-old woman presents with complaints of shortness of breath and severe fatigue for over two weeks. There was no history of vomiting or hematemesis, no obvious blood in the stools, and no abdominal pain. Her appetite had been normal. She had a history of a recent upper respiratory infection with fever, which was treated with oral antibiotics and decongestants. Her past medical history indicated frequent intake of non-steroidal anti-inflammatory drugs for osteoarthritis for many years. She had been taking over-the-counter antacids and proton pump inhibitors (PPIs) intermittently for gastroesophageal reflux disease concomitantly. Two years prior to this episode, she had experienced similar symptoms and was observed to be very pale and weak at that time. The hemoglobin had been 6.5 g/dl, and she had a very low serum iron level. Her hemoglobin had improved to 12.8 g/dl with iron tablets and dietary changes. Investigations such as CXR or endoscopy were not done at that point as she clinically improved with oral medication. She had no history of hypertension or diabetes. Renal and other metabolic profiles had been normal. She had a surgical history of a hysterectomy and L3-L5 laminectomy with spinal fusion done 15 years earlier.

**Figure 1:**
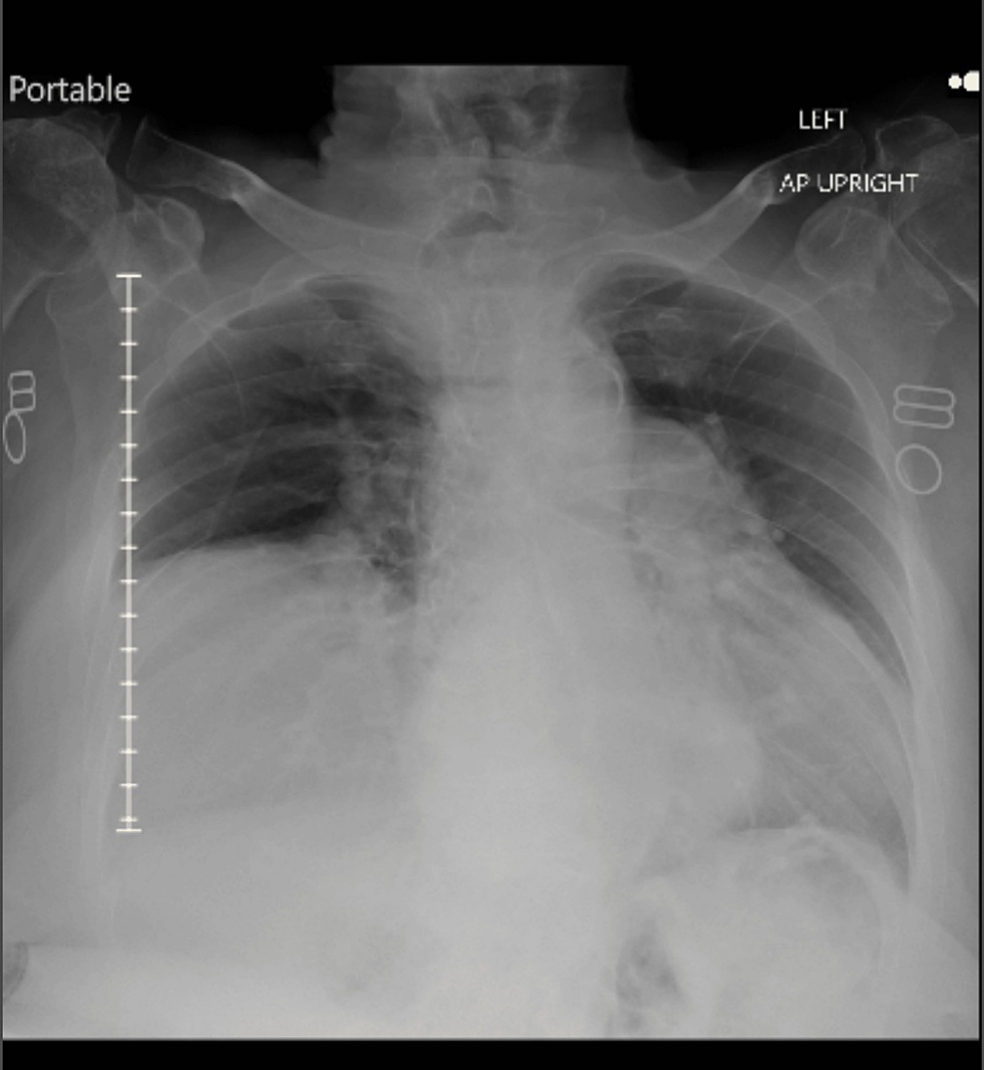
Plain chest X-ray (CXR) of the patient showing limited lung capacity, moderate cardiomegaly, and a soft tissue shadow surrounding the heart on the left

Physical examination during the current presentation showed severe pallor of the conjunctiva, pedal edema, tachypnea, and kyphosis. Lungs were generally clear, and a remnant cough from a recent viral illness was observed. Heart sounds were normal. There were no murmurs and no gallop. The abdomen was soft, non-tender, and had no hepatosplenomegaly. Her blood pressure was 106/78, her heart rate was 99 beats/min, and her respiratory rate was 22/min. Laboratory investigations showed a hemoglobin level of 5.6 g/dl, low reticulocyte count, a decrease in red cell distribution width, and a low serum ferritin level. A peripheral blood smear evidenced microcytic hypochromic anemia. Renal and metabolic profiles were normal. The stool was positive for occult blood. The performed electrocardiogram was normal. A CXR showed mild cardiomegaly with an elevated right hemidiaphragm. A soft tissue shadow was observed around the heart within the chest cavity, which was suggestive of HH (see Figure 1). A CT scan showed a large paraesophageal HH containing most of the stomach, which demonstrated an upside-down configuration. Moderate cardiomegaly was noted. The middle lobe of the right lung exhibited sub-segmental atelectasis.

**Figure 2:**
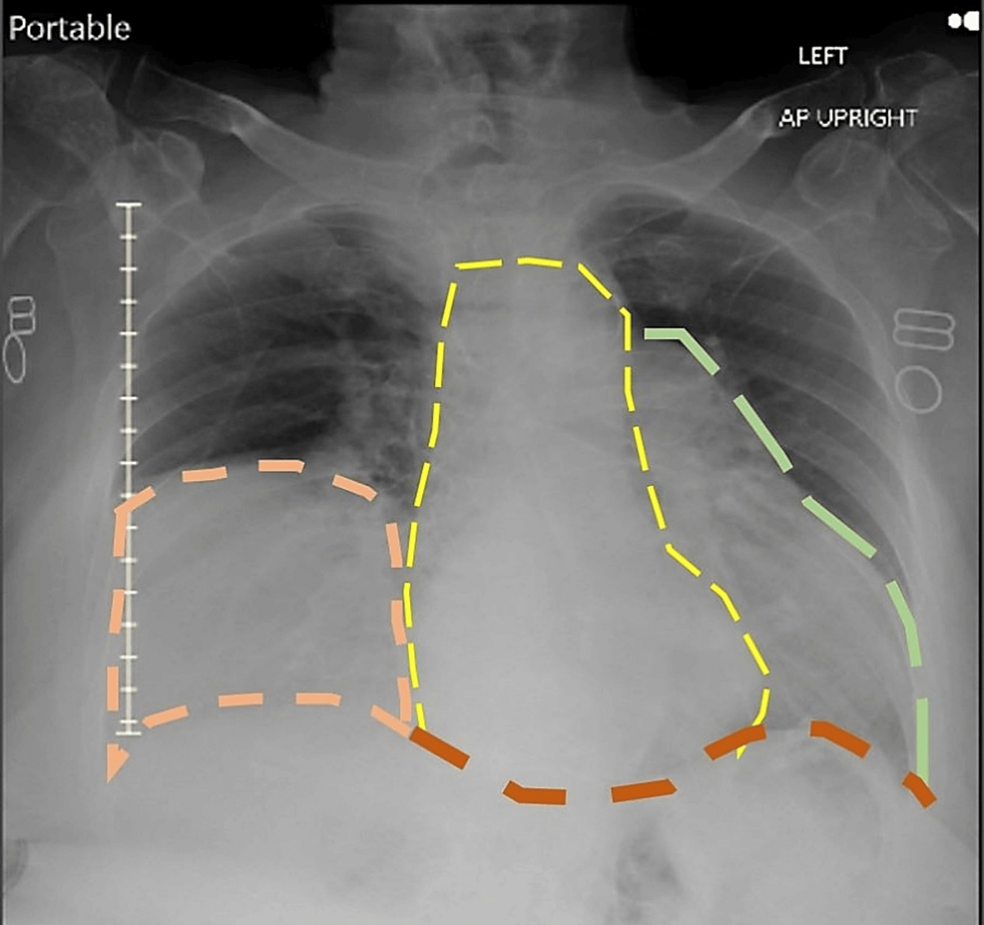
The patient's chest X-ray (CXR) The diaphragm is indicated by an orange to the red dotted line from the right side of the chest to the left; the yellow dotted line indicates the heart and the green dotted line outlines the herniated stomach. The orange dotted line circumscribes the elevated right dome of the diaphragm and the atelectasis of the middle lobe of the right lung.

Figure 1 shows the plain radiograph with the observations as reported above. The patient's CXR reveals moderate cardiomegaly and an abnormal soft tissue shadow around the cardiac silhouette, which is suggestive of HH. The middle lobe of the right lung shows atelectasis. Figure 2 shows the abnormalities with dotted lines in different colors.

**Figure 3:**
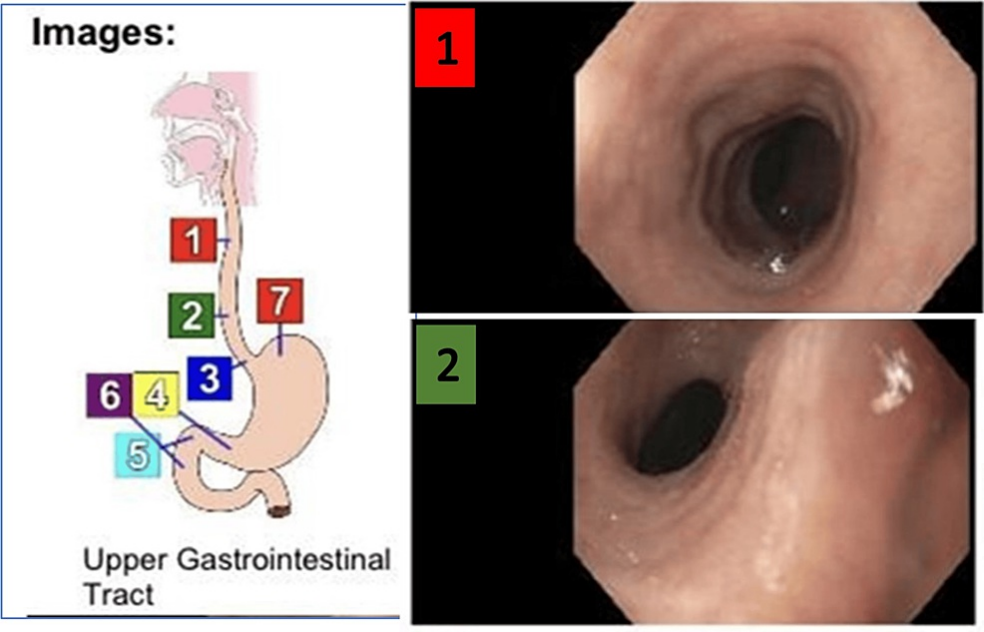
Esophagogastroduodenoscopy images A series of images visualizing the areas as indicated in the picture on the left (areas where the images are taken are indicated with various colors). 1: the image was taken at the middle one-third of the esophagus. 2: the image was taken at the lower third of the esophagus.

**Figure 4:**
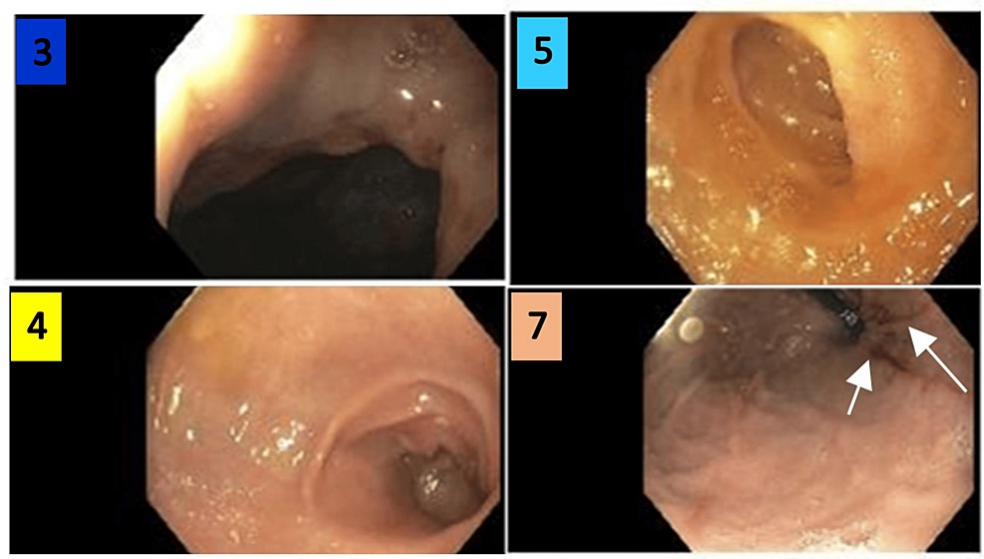
Esophagogastroduodenoscopy images 3: the image was taken at the cardio-esophageal junction. 4: the image was taken at the pre-pyloric stomach. 5: the image was taken at the duodenal bulb. 7: the image was taken at the gastric body; white arrows indicate Cameron lesions within the hiatus hernia.

**Figure 5:**
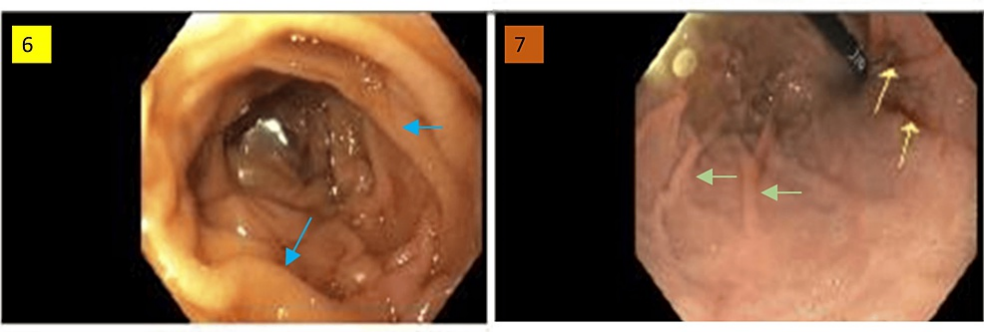
Esophagogastroduodenoscopy images 6: the image shows a normal second portion of the duodenum; blue arrows indicate the plicae circulares. 8: the image shows Cameron lesions within the stomach, indicated by yellow arrows; the green arrows indicate the rugae of the stomach.

EGD showed a large paraesophageal HH, which was reported to be more than six cm in diameter. Gastric erosions, in this case, Cameron lesions, were found without any stigmata of bleeding in the cardiac region. The upper, middle, and lower segments of the esophagus were normal. The mucosa of the duodenum appears to be normal. The images taken at the respective levels during the EGD are shown in Figures 3-5. A diagram shown in Figure 3 shows the levels where the images were captured, indicated with colored labels. 

Figure 4 shows images taken from the cardio-esophageal junction, pre-pyloric region of the stomach, and within the duodenum. An image within the herniated segment of the stomach showing the Cameron lesions is also shown here.

Figure 5 shows images that were taken during EGD. The image on the left indicates the duodenum showing normal and healthy mucosa lining the plicae circulares. The stomach shows Cameron lesions within the mucosa. The rugae, indicated by green arrows, confirm the image of the stomach. 

As for the treatment, two units of packed cell transfusion were administered, along with parenteral iron. The patient was discharged on oral iron, stool softeners, and PPIs. Dietary changes were advised. Follow-up hemoglobin in 10 days was 10 g/dl. The patient had clinically improved over two weeks of treatment and was scheduled for a follow-up visit in six weeks.

## Discussion

**Figure 6:**
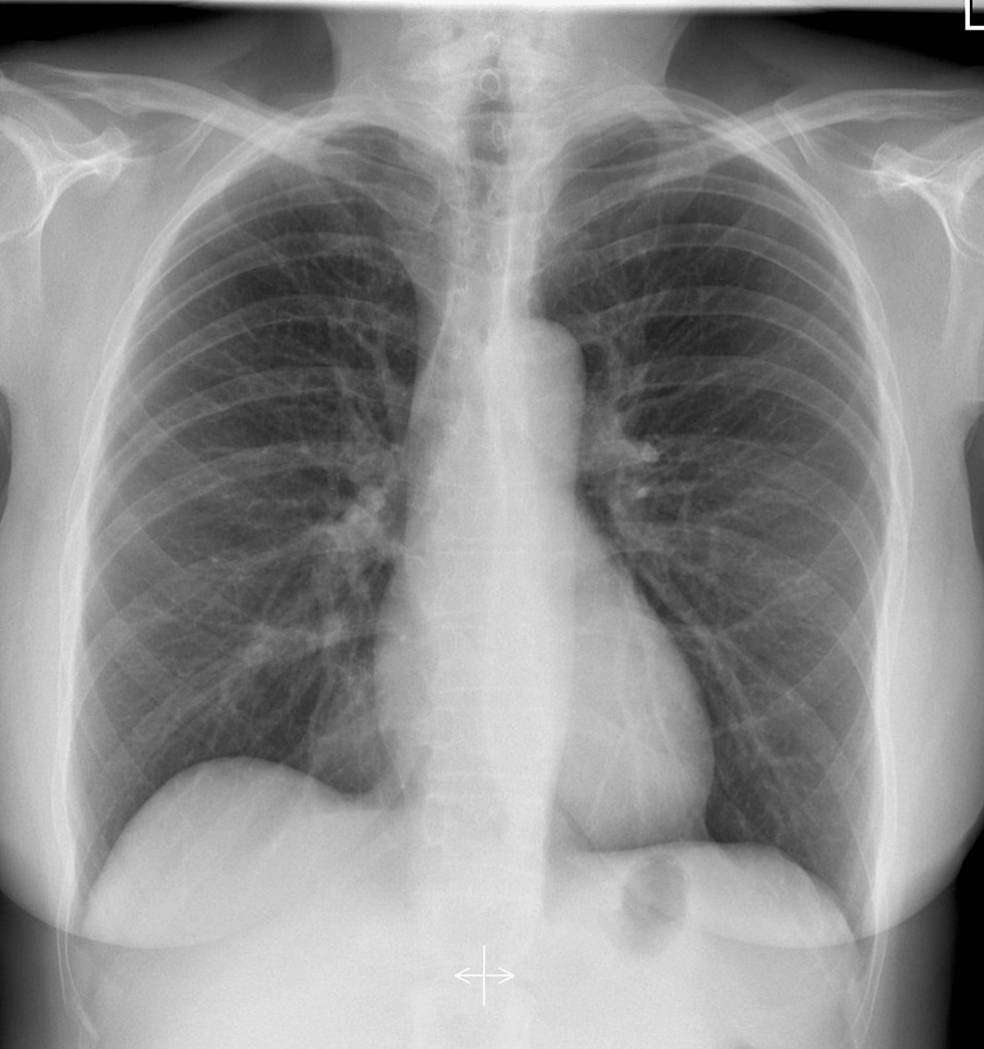
A normal chest X-ray (CXR) for comparison The trachea can be identified in the midline. The position of the diaphragm is normal. Lungs and pleural spaces are clear. Cardio mediastinal contour is normal. This image is reproduced under Creative Commons license [3].

Figure 6 shows a CXR of an adult female that shows the normal location of the diaphragm, the normal size of the heart, and normal shadow and pleural spaces [3]. The right hemidiaphragm is mildly elevated than the left due to the underlying liver, which is generally a normal finding. This image is presented here to compare it with the image performed in our case study.

Based on the normal image shown above, our patient's CXR revealed an enlarged heart with a soft tissue shadow surrounding the heart, which is suggestive of HH. The elevated right diaphragm with a report of middle lobe atelectasis of the right lung was clinically correlated to the recent lower respiratory infection. This infection could have been the cause of severe and profuse cough, which could be a factor responsible for raised intraabdominal pressure resulting in the herniation. In the case of a preexisting HH, this infection could have been an aggravating factor. 

Microcytic hypochromic anemia is a common finding in peripheral smears due to iron deficiency anemia. The common causes of this anemia are decreased intake, poor absorption, blood loss, and increased iron requirements. In postmenopausal women, the frequent cause of blood loss is chronic occult bleeding, usually from the gastrointestinal tract, for example, due to peptic ulcer disease, malignancy, hemorrhoids, or vascular ectasias. Causes of decreased iron absorption can result from gastrectomy or malabsorption syndromes, such as celiac disease, atrophic gastritis, Helicobacter pylori infection, achlorhydria, short bowel syndrome, and iron-refractory iron deficiency anemia [4]. The common symptoms of anemia include fatigue, loss of stamina, shortness of breath, weakness, dizziness, and pallor. Patients may or may not present themselves to the physician immediately, and it may depend on the rate of blood loss [4]. In relation to this case presentation, the treatment started with transfusions to improve the general condition and stabilize the patient, and then investigations followed. Based on the findings of Cameron lesions within the HH, the cause of bleeding needs to be discussed.

Erosive lesions in the stomach have been described since the early 1970s but are credited to Cameron and Higgins, who described them in detail in 1986 in a study of 109 patients with gastric erosions [5]. They found that many of them exhibited anemia, and most of those with anemia showed the presence of large HH. It was reasoned that these erosions were due to trauma, and the microcytic hypochromic anemia resulted from blood loss. It is reported in the literature that five percent of all endoscopies have identified Cameron lesions in HHs. Following the description of these lesions in detail, more cases were reported over the past 30 years. In 1996, studies done in Kansas City, Missouri, indicated an incidence of Cameron lesions in 5.2% of cases with large HHs [6]. These studies indicated that the presence of these lesions is incidental in EGDs and may not always be associated with blood loss. Most cases had an acid peptic disease, reflux esophagitis, and associated complications. Mucosal injury due to mechanical trauma, acid, and ischemia can be the leading cause of Cameron lesions. It is crucial to note that of the patients who underwent medical therapy, one-third had a recurrence of lesions, and 17% had developed complications, such as acute UGIB or persistent and recurrent iron deficiency anemia. In 2008, Maganty and Smith presented a case of UGIB with HH and Cameron lesions [7]. The patient presented with low hemoglobin and was given a transfusion, following which the bleeding stopped spontaneously. The patient was discharged on famotidine. However, she returned with bleeding two months later. They also report that histopathology done from several biopsies of Cameron lesions showed focal ischemic gastropathy consisting of coagulative necrosis. Panzuto et al. [8] and Pauwelyn and Verhamme [9] conducted extensive studies on patients with large HH and demonstrated that such hernias can cause iron deficiency anemia even without Cameron lesions. While some studies say that they are the primary cause of UGIB in patients with HH, some others indicate that the presence of Cameron lesions is not a necessity in such hernias.

Ruhl and Everhart conducted a population-based study [10] in 2001 to see the relationship between iron deficiency anemia in patients with isolated large HH, isolated esophagitis, and patients with both diagnoses. They concluded that patients with HH were hospitalized subsequently with severe iron deficiency anemia. Whereas the relationship between esophagitis and iron deficiency anemia requires further evaluation.

The treatment options for this condition include medical management, surgery, and endoscopic intervention. Endoscopic band ligation in actively bleeding lesions has shown good results in a limited number of cases. Cauterization and epinephrine injection have been tried as temporary measures. The surgical option of fundoplication is recommended in patients with uncontrolled bleeding who are refractive to medical management [1].

## Conclusions

Based on the available information from the literature and from this case presentation, if a patient presents with signs of severe anemia, with a CXR showing suspicion of HH, a strong consideration of Cameron lesions being the primary etiology should be considered. Findings of microcytic hypochromic type of anemia and a positive EGD for Cameron lesions in these patients can serve as confirmatory findings. Generally patients improve with the treatment with proton pump inhibitors, oral and parenteral iron, within six weeks. If the symptoms are persistent after this period, there is still a need to explore and resolve aggravating underlying conditions. Once that is achieved and if the patient is fit for surgical procedure, fundoplication may be offered.
